# Immune Tolerance in *Mytilus galloprovincialis* Hemocytes After Repeated Contact With *Vibrio splendidus*

**DOI:** 10.3389/fimmu.2019.01894

**Published:** 2019-08-09

**Authors:** Magalí Rey-Campos, Rebeca Moreira, Marco Gerdol, Alberto Pallavicini, Beatriz Novoa, Antonio Figueras

**Affiliations:** ^1^Institute of Marine Research (IIM), CSIC, Vigo, Spain; ^2^Department of Life Sciences, University of Trieste, Trieste, Italy; ^3^Istituto Nazionale di Oceanografia e di Geofisica Sperimentale – OGS, Trieste, Italy

**Keywords:** *Mytilus galloprovincialis*, *Vibrio splendidus*, hemocyte, RNA-Seq, immune priming, ROS, inflammation, apoptosis

## Abstract

Mediterranean mussels (*Mytilus galloprovincialis*) are sessile filter feeders that live in close contact with numerous marine microorganisms. As is the case in all invertebrates, mussels lack an adaptive immune system, but they respond to pathogens, injuries or environmental stress in a very efficient manner. However, it is not known if they are able to modify their immune response when they reencounter the same pathogen. In this work, we studied the transcriptomic response of mussel hemocytes before and after two consecutive sublethal challenges with *Vibrio splendidus*. The first exposure significantly regulated genes related to inflammation, migration and response to bacteria. However, after the second exposure, the differentially expressed genes were related to the control and inhibition of ROS production and the resolution of the inflammatory response. Our results also show that the second injection with *V. splendidus* led to changes at the transcriptional (control of the expression of pro-inflammatory transcripts), cellular (shift in the hemocyte population distribution), and functional levels (inhibition of ROS production). These results suggest that a modified immune response after the second challenge allowed the mussels to tolerate rather than fight the infection, which minimized tissue damage.

## Introduction

*Mytilus galloprovincialis* is widely distributed throughout the world and has a high ecological and economic impact (1). Due to their status as sessile and filter-feeding animals, bivalves are exposed to a continuous stream of microorganisms, some of them pathogens (2), and environmental pollutants (3). Consequently, mussels have been used as sensors in ecotoxicological studies to monitor the quality of the marine environment (4, 5). Bivalves, such as *Crassostrea gigas*, are susceptible to diseases that may cause massive mortality (6, 7). Surprisingly, despite living in the same ecosystem and being exposed to the same pathogens, no significant mortality in *M. galloprovincialis* has been reported (8, 9).

Although lacking acquired immune response, hemocytes, which are the immune cells in bivalves, respond to pathogens with chemotaxis, encapsulation, phagocytic activity and the release of oxygen and nitrogen radicals (10). Moreover, hemocytes can recognize pathogen-associated molecular patterns (PAMPs) via pattern recognition receptors (PRRs) to activate intracellular signaling pathways to finally trigger the synthesis of antimicrobial effectors (11, 12). In this sense, transcriptomic information regarding the modulation of the hemocyte immune response in bivalves remains scarce (13–17). In mussels, several genes related to key immune functions have been characterized over the past few years. These include different molecules involved in specific pathogen recognition, such as C-type lectins (18), C1q domain-containing proteins (19), and proteins with a fibrinogen-related domain (FReD) (20). Compared to other bivalves, mussels are also particularly rich in antimicrobial peptides (AMPs), and myticin C is an example of an important immune effector with chemotactic, antibacterial and antiviral activities (21, 22). Lysozyme, which is able to hydrolyze the central components of the bacterial wall, is another key player in the effector arm of the mussel immune response (23). Gerdol and Venier (24) have reviewed the presence and the interplay between the different molecular components of the mussel immune defense system by using information found in public sequence databases.

In recent years, some studies have suggested that invertebrates may respond to an infection due to some degree of innate immune memory or “priming.” Because of this, exposure to a non-lethal dose of a pathogen could provide protection against later infection with the same pathogen (10, 25). Several studies have recently reported the protection of oysters (*Crassostrea gigas*) from subsequent infection with ostreid herpesvirus (OsHV-1) by the use of poly I:C as a priming effector (26–28).

The main objectives of the present study were to characterize the transcriptomic and functional response of mussel hemocytes after injection with *Vibrio splendidus*, which has been reported to produce mortality in mussels (9), and to analyze whether a different type of response could be elicited after a second interaction with the same pathogen. The outcome of this experimental approach might help to reveal the trainability of the mussel immune response and to identify genes associated with this process.

## Materials and Methods

### Animals

Adult *M. galloprovincialis* with shells 8–10 cm in length were obtained from a commercial shellfish farm (Vigo, Galicia, Spain) and maintained in open circuit filtered seawater tanks at 15°C with aeration. The animals were fed daily with *Phaeodactylum tricornutum* and *Isochrysis galbana*. Prior to the experiments, the animals were acclimatized to aquarium conditions for 1 week.

### Experimental Approach

Twenty mussels were marked and notched in the shell, and hemolymph (500 μl) was withdrawn from the adductor muscle of each mussel with a 0.5 mm diameter (25 G) disposable needle. The hemolymph sampled at time zero (t0) was centrifuged at 4°C at 3,000 g for 10 min, and the pellet was resuspended in 500 μl of TRIzol (Invitrogen), immediately homogenized and stored at −80°C until RNA isolation.

After 1 week, 10 mussels were injected in the adductor muscle with 100 μl of filtered seawater (FSW). The other 10 mussels were injected in the same way with 100 μl of a solution of *V. splendidus* (reference strain, LGP32) at a non-lethal concentration (1 × 10^7^ UFC/ml). One day post injection (24 hpi) and 7 days post injection, hemolymph (500 μl) was sampled again from individual mussels and centrifuged in the same conditions, and the pellet was resuspended in 500 μl of TRIzol (Invitrogen). Samples were immediately homogenized and kept at −80°C until RNA isolation.

After 2 weeks, the 10 mussels injected with FSW were injected again with FSW. The mussels previously exposed to *V. splendidus* were injected again with a solution of *V. splendidus* (reference strain, LGP32) at a non-lethal concentration (1 × 10^8^ UFC/ml). One day after the second injection (24 hpi2), hemolymph (500 μl) was sampled again from individual mussels and centrifuged in the same previously described conditions, and the pellet was resuspended in 500 μl of TRIzol (Invitrogen). The samples were immediately homogenized and kept at −80°C until RNA isolation.

Seven days later (7 d), hemolymph (500 μl) was sampled again from the mussels and centrifuged in the same previously described conditions, and the pellet was resuspended in 500 μl of TRIzol (Invitrogen). The samples were immediately homogenized and kept at −80°C until RNA isolation.

### *Vibrio splendidus* Clearance Assessment

The clearance of *V. splendidus* was assessed to make sure that the second injection was made after a complete overcome of a possible infection. cDNA was synthesized from samples taken at t0, 24 hpi, and 7 days after the first infection with 100 ng of total RNA using an NZY First-Strand cDNA Synthesis Kit (nzytech). Gene expression of *V. splendidus* 16S and mussel 18S (used as a reference gene) was analyzed in a Stratagene Mx3005P thermal cycler (Agilent Technologies). In addition, ten-fold dilutions of *V. splendidus* DNA was included in the plate to extrapolate the bacterial load of each sample.

For 16S detection, 5 μl of five-fold-diluted cDNA template was mixed with 0.6 μl of each primer (10 μM), 0.4 μl of 16S probe (10 μM) and 10 μl of Brilliant III Master Mix 2x Ultrafast (Agilent Technologies) in a final volume of 20 μl. For 18S detection 1 μl of five-fold-diluted cDNA template was mixed with 0.5 μl of each primer (10 μM) and 12.5 μl of Brilliant II SYBR Green (Agilent Technologies) in a final volume of 25 μl. The standard cycling conditions were 95°C for 10 min, followed by 40 cycles of 95°C for 15 s and 60°C for 30 s. All reactions were performed as technical triplicates. The relative expression levels of the genes were normalized using 18S as a reference gene following the Pfaffl method. One-way ANOVA was used to analyze differences in normalized gene expression and bacterial load among the studied samples.

### RNA Isolation, cDNA Production, and Illumina Sequencing

RNA isolation was carried out using TRIzol (Invitrogen) according to the manufacturer's protocol. RNA purification was performed after DNase I treatment with the RNeasy Mini Kit (Qiagen). Next, the concentration and purity of the RNA were measured using a NanoDrop ND1000 spectrophotometer, and the RNA integrity was verified with an Agilent 2100 Bioanalyzer (Agilent Technologies). Only the best RNA samples (in terms of RNA quantity and quality) from four sampling points (t0, 24 hpi, 24 hpi2, 7 d) were chosen for the preparation of cDNA libraries compatible with Illumina sequencing. The chosen samples were from the mussels control 2, control 3, control 4, infected 1, infected 2 and infected 10. A total of 24 samples were selected for sequencing that consisted of 2 conditions, 4 sampling points and 3 biological replicates. Some of the samples corresponding to 7 days after the first injection were not of sufficient quantity to be sequenced, and therefore this sampling point was excluded from the analysis.

A TruSEq library preparation kit from Illumina was used according to the manufacturer's instructions. Briefly, eukaryotic mRNA was extracted from total RNA using oligo (dT) magnetic beads and cleaved into short fragments using fragmentation buffer. A cDNA library was then prepared from the fragmented mRNA via reverse transcription, second-strand synthesis and the ligation of specific adapters (paired-ends) after cDNA purification using the QIAquick PCR Purification Kit (Qiagen). The amount of cDNA in each library was quantified through spectrofluorometric analysis using the Qubit system. Paired-end sequencing (2 × 100) was performed using an Illumina HiSeq™ 4000 platform by Macrogen Korea.

**Figure 1:**
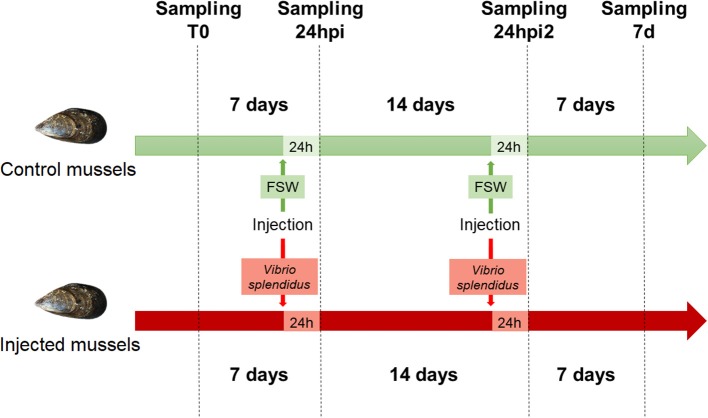
Experimental design used for the stimulation of mussels.

A schematic representation of the experimental design for the sequenced samples is shown in Figure 1. The raw sequencing data have been deposited in the NCBI Short Read Archive database under the accession ID SRP145077.

### Bioinformatics and RNA-Seq

The CLC Genomics Workbench, v.11.0.1 (CLC Bio; Qiagen), was used to process the raw sequencing output for the *de novo* assembly of the reference transcriptome and to perform the statistical analyses of gene expression by comparing the three biological replicates for the control and infected mussels at different time points. The raw reads were trimmed to remove adaptor sequences, low quality bases (quality score *p*-value limit of 0.05), and residual sequences shorter than 70 bp. All reads obtained from the 24 libraries were assembled to obtain a complete reference transcriptome with default *word size* and *bubble size* parameters. The assembly was cleaned to remove sequences originating from mussel ribosomal RNA and mitochondrial mRNAs, as well as contaminant transcripts from *V. splendidus*, ciliates and microalgae. These filtering steps were performed with BLASTn analyses (e-value threshold 1e-10) that were carried out in parallel with the reported assembled mussel genome (29) and the targets mentioned above (the *Vibrio splendidus* genome from strain NCCB 53037, the ciliate *Pseudocohnilembus persalinus* genome, the *Phaeodactylum tricornutum* genome and the *Isochrysis galbana* transcriptome from BioProject PRJNA248394 were used as references). Contigs that produced a more significant hit when compared to the sequences of the putative contaminants than to the mussel genome were discarded. The quality and completeness of the transcriptome were assessed with BUSCO v.3 (30), which was based on the detection of metazoan Benchmarking Universal Single Copy Orthologs (BUSCOs) according to release 9 of OrthoDB.

The reads of each individual mussel and sampling were mapped onto the clean transcriptome with the RNA-Seq tool using the following parameters: mismatch cost = 2, length fraction = 0.8, similarity fraction = 0.8, and maximum hits per read = 10. Differentially expressed genes (DEGs) were identified with a statistical analysis based on generalized linear models and by assuming a negative binomial distribution for the read counts (31). For each set of comparisons, transcripts with an absolute fold change (FC) value > 2 and an FDR-corrected *p* < 0.05 were considered differentially expressed and retained for further analyses. To find the DEGs at each time point after the *Vibrio* challenges, the injected samples were compared with their respective controls. To find primed and tolerized genes, the *Vibrio* challenged samples from the second injection were compared with the challenged samples from the first injection, and the same comparison was made in the controls to confirm that the selected genes were not modulated in control animals.

### BLAST Annotation, GO Assignments, and Enrichment Analysis

The transcriptome was functionally annotated with the Blast2GO software (32) by assigning gene ontology (GO) terms based on the significant BLASTx matches found in the UniProt/Swissprot database. To improve the annotation rate, we performed an additional BLASTn analysis against an in-house database, which included all the molluscan sequences present in the NBCI nucleotide database. In both cases, the e-value threshold for annotation was set to 1e-3. Then, functional enrichment analyses of the DEGs (test set) were conducted using the full mussel transcriptome as the reference set. For this purpose, a two-tailed Fisher's exact test was performed with the default parameters and a *p*-value cut-off of 0.05. The test was performed on the basis of overrepresented biological process (BP) gene ontology terms.

### Functional Assays: Hemocyte Distribution, Apoptosis, and ROS Analyses

The previously described experimental design was repeated using eight biological replicates (each replicate included a single mussel) to determine whether functional immune parameters were also affected by a second exposure to the same bacterial pathogen. Hemolymph was collected from the adductor muscle of the eight individual mussels using a disposable syringe, and the cell concentration was adjusted to 10^6^ cells ml^−1^ with FSW.

The hemocyte populations were evaluated by flow cytometry. Two FSC/SSC gates were created that included both the viable granulocyte and the hyalinocyte populations. Data were acquired using a FACS Calibur flow cytometer (Becton and Dickinson), and the analysis was carried out using CellQuest software (Becton and Dickinson).

To investigate the possibility that the mussels that received a single and two subsequent injections of *V. splendidus* showed changes in cell death rates, an apoptosis analysis was performed. Hemocytes were centrifuged and resuspended in 1 ml of binding buffer (BB1X). Then, 5 μl of annexin V (Invitrogen) and 5 μl of actinomycin (BD Pharmingen) were added to the cell suspensions. The samples were incubated for 15 min at room temperature in the dark and analyzed by flow cytometry.

The respiratory burst activity of hemocytes was determined by the luminol-enhanced chemiluminescence method (CL) in 96-well-plates. We used 5-amino-2,3-dihydro-1,4-phthalazinedione (luminol, Sigma Aldrich) as a light emitter and phorbol myristate acetate (PMA, Sigma Aldrich) or zymosan A (Sigma Aldrich) to trigger the production of reactive oxygen species (ROS). A stock solution of 0.1 M luminol was prepared in dimethyl sulphoxide (DMSO, Sigma Aldrich) and diluted in FSW to obtain the luminol working solution (final concentration of 10 mM). Zymosan A (20 mg ml^−1^) was diluted in the luminol working solution to obtain a final concentration of 1 mg ml^−1^. The PMA stock solution (1 mg ml^−1^ in ethanol) was also diluted in the luminol working solution to obtain a final concentration of 1 μg ml^−1^.

One hundred microliters of hemolymph was dispensed into each well of the 96-well-plates. After 30 min of incubation at 15°C, 100 μl of luminol, PMA or zymosan A were added per well. The relative luminescence units (RLU) were measured in a luminometer (Fluoroskan Ascent, Labsystems) six times at intervals of 5 min with an integration time of 1,000 ms for each measurement.

## Results

### Assembly and Annotation of the Mussel Transcriptome

The sequencing of the individual hemocyte samples yielded an average of 74.11 million raw reads for each of the 24 libraries. The trimming procedure removed, on average, 0.83% of the raw reads, and a total of 1,778 million reads were assembled into a reference mussel transcriptome containing 260,664 contigs with an average length of 512 bp. The reference transcriptome was highly complete, as just 2% of metazoan BUSCOs were absent, and displayed a fragmentation rate equal to 22%, which was in line with what was expected for such a highly heterozygous species (Table 1).

**Table 1:** Summary of the transcriptome bioinformatics pipeline.

**Sample**	**Raw**	**Trimmed**
C2 t0	78,426,948	99.59%
C3 t0	44,346,854	98.03%
C4 t0	100,814,198	99.59%
I1 t0	17,696,894	98.08%
I2 t0	93,114,098	99.60%
I10 t0	96,780,602	99.64%
C2 24h	95,296,484	99.49%
C3 24h	51,708,988	97.62%
C4 24h	92,661,282	99.65%
I1 24h	52,102,302	98.77%
I2 24h	90,965,875	99.52%
I10 24h	99,262,970	99.55%
C2 24h2	93,665,372	99.96%
C3 24h2	47,632,114	99.09%
C4 24h2	83,629,996	99.62%
I1 24h2	25,833,124	98.25%
I2 24h2	90,636,926	99.60%
I10 24h2	101,411,946	99.64%
C2 7d2	78,528,396	99.55%
C3 7d2	46,511,774	99.36%
C4 7d2	95,180,866	99.69%
I1 7d2	13,593,322	97.43%
I2 7d2	90,614,296	99.31%
I10 7d2	98,236,250	99.57%
**Assembly statistics**		
Contigs	260,664	
Range contig length	200–15,624	
Average contig length	512	
N50	576	
Complete metazoan BUSCOs	740/978 (75.69%)	
Fragmented metazoan BUSCOs	218/978 (22.29%)	
Missing metazoan BUSCOs	20/978 (2.04%)	
**Blast**		
Contigs with hit in Uniprot/SwissProt	19.93%	
Contigs with hit in mollusks database	42.64%	
**GO analysis**		
Annotated contigs	23.35%	
**KEGG analysis**		
Pathway assigned contigs	7.27%	

Two different BLAST approaches were used to annotate the assembled transcriptome. In brief, 42.64% of the contigs were found to have a significant match in the custom database, which included all the mollusk nucleotide sequences available from NCBI, and Blast2GO was used to annotate 19.93% of the contigs through a BLASTx search of UniProt/SwissProt (Supplementary File 1). Based on these results, gene ontology (GO) terms were assigned to 23.35% of the contigs. Table 1 shows the sequencing output of all the samples and the main metrics of the transcriptome assembly and annotation.

### Transcriptomic Response After Injection and Reinjection With the Same Pathogen

**Figure 2:**
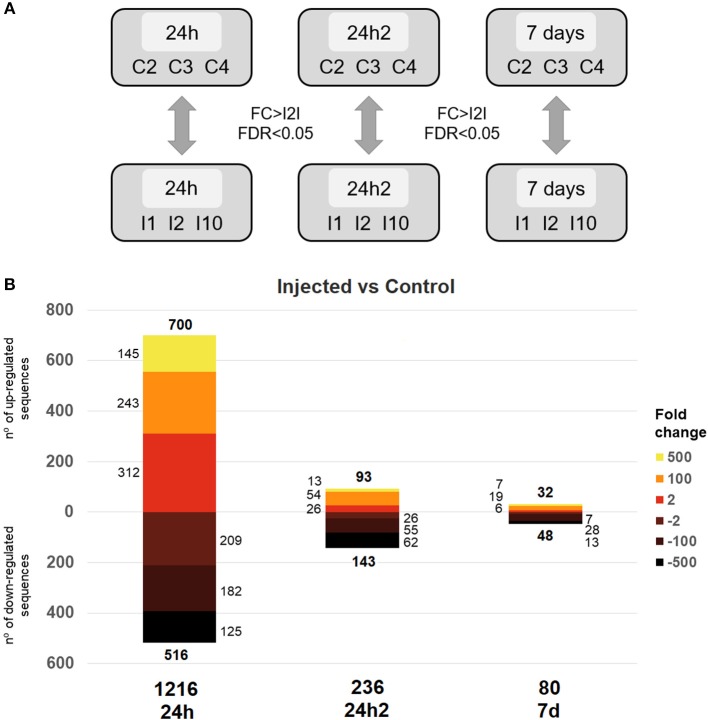
**(A)** Representative scheme of the comparisons made for the differential expression analysis between injected and control individuals at the analyzed sampling points. The thresholds used to detect statistical significance were a fold change (FC) > |2| and an FDR < 0.05. **(B)** Distribution of the response magnitudes of the DEGs. Statistically significant gene modulation is shown according to intensity (fold-change) and direction (up- and down-regulation).

We carried out a differential gene expression analysis to gain insights into the dynamics of the transcriptional response of mussel hemocytes to an experimental infection with *V. splendidus*. First, the clearance of *V. splendidus* by the injected mussels was confirmed 7 days after the first injection: *V. splendidus* detection increased 24 hpi and was rapidly controlled 7 days after the injection, returning to control levels (Supplementary File 2). To analyze the transcriptomic response at each sampling point, the *Vibrio* injected animals were compared to control animals (FSW-injected) (Figure 2A). The monitoring of the transcriptional profiles enabled us to assess whether a second interaction with the same pathogen could elicit a different type of response compared to the response elicited by the first injection. A total of 1,216 differentially expressed genes (DEGs) were detected 24 h after the first injection (24 hpi). However, this number dramatically decreased to 236 DEGs 24 h after the second injection (24 hpi2) and dropped further to 80 DEGs when the transcriptional profiles were compared 7 days after reinjection (7 d) (Figure 2B).

**Figure 3:**
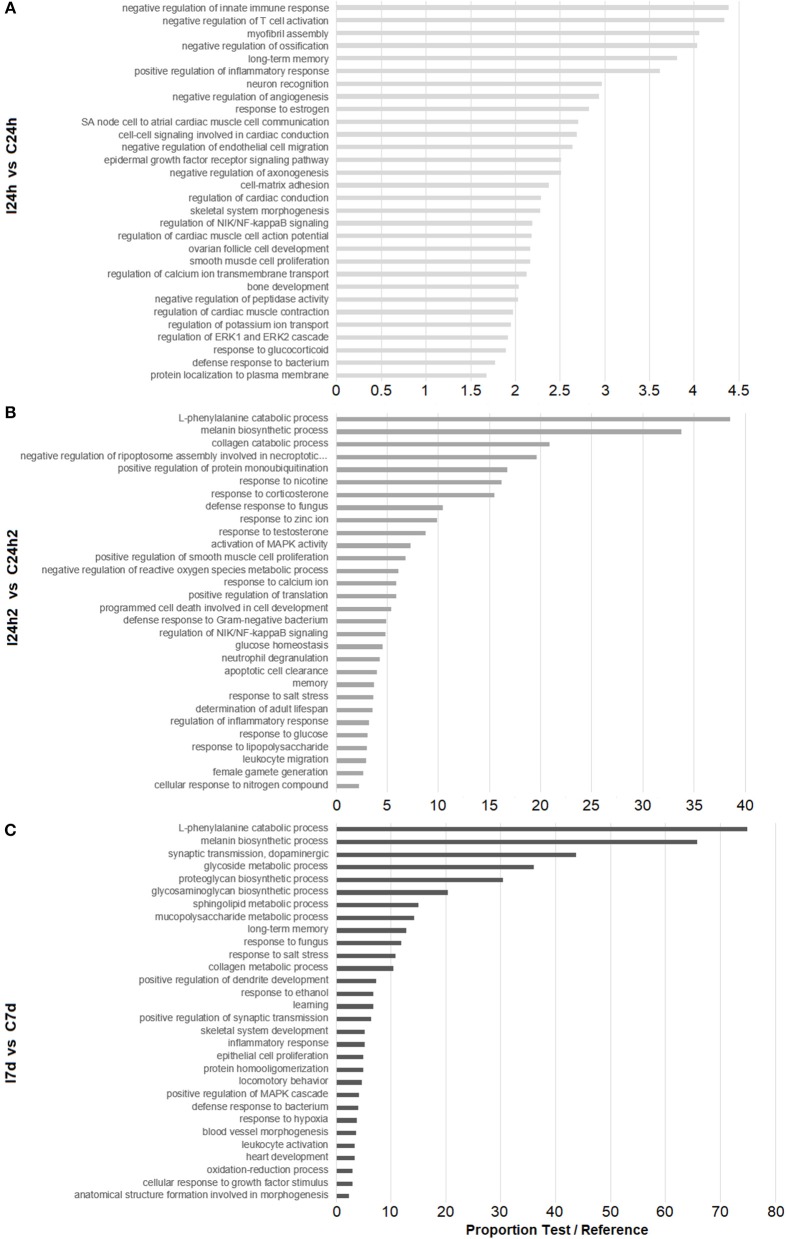
Enrichment analysis of DEGs according to the experimental design. Bars represent the proportions between the percentages of sequences in the test set (our DEGs list) and the reference set (transcriptome). **(A)** Biological processes overrepresented in infected mussels 24 h after the first infection. **(B)** Biological processes overrepresented 24 h after the second infection. **(C)** Biological processes overrepresented 7 d after the second infection.

To detect any significant alteration in biological pathways, a Fisher's exact test was performed. An enrichment analysis of the GO annotations associated with the DEGs at each sampling point (24 hpi, 24 hpi2, and 7 d) was conducted. The 30 most significantly enriched GO terms for each sampling point are shown in Figure 3. After the first injection (24 hpi), GO terms related to the immune system were found, such as those related to the regulation of innate immune response, inflammatory response, cell migration and defense response to bacteria. After the second injection (24 hpi2), genes related to the inflammatory response seemed to also be modulated and were represented in processes involved in the regulation of the NF-kB signaling pathway. Moreover, processes involved in defense response to bacteria and fungi, the negative regulation of ROS, apoptosis and glucose homeostasis appeared to be regulated after reinjection. The last sampling point (7 d) showed a modulation of genes related to GO terms involved in neural processes (long-term memory and learning), tissue regeneration (cell population proliferation and proteoglycan, glycosaminoglycan, mucopolysaccharide, and collagen metabolism) and the resolution of infections (oxidation-reduction processes and defense response to pathogens).

The most highly expressed genes at each sampling point are shown in Table 2 (complete information in Supplementary File 3). After the first exposure, several genes showed high expression values and were significantly decreased as the experiment progressed (reinjection and 7 d). This was the case for perlucin-like protein, which is directly involved in pathogen recognition, the spore cortex-lytic enzyme, which can destroy the bacterial cell wall, and the henna protein, which is important for melanization; all of these genes could play crucial roles in the killing and sequestration of invading pathogens. These genes reached very high fold-change values after the first injection, and they decreased after reinjection and exhibited their lowest values 7 days after reinjection. Other interesting genes related to recognition (lectin, neurocan, and galaxin), acute phase response (HSP70 and sacsin), antimicrobial response (apextrin) and apoptosis (caspase 3 and the GTPase IMAP family member 4) were up- or down-regulated in a balanced manner (15 up- and 10 down-regulated). However, 1 day after the second challenge, the majority of the most highly regulated genes were down-regulated (23 down- and 2 up-regulated). For example, antimicrobial peptides such as defensin MGD-1 or myticin B were not differently regulated after the first injection, but they were indeed inhibited after reinjection (FC −1,360) and 7 days after the second injection (FC −3,745) (Supplementary File 3).

**Table 2:** Top 25 DEGs at each sampling point. FC, fold change.

**I24h vs. C24h**		**I24h2 vs. C24h2**		**I7d vs. C7d**	
**FC**	**Description**	**FC**	**Description**	**FC**	**Description**
−6068.31	Caspase-3	−20257.80	Vitellogenin	−3745.45	Myticin B
−5928.63	Neuronal acetylcholine receptor subunit alpha-2	−7278.01	TRPM8 channel-associated factor 2	−1031.26	Nacre apextrin-like protein 1
−5633.81	Metalloproteinase inhibitor 3	−5276.43	Latrophilin Cirl	−956.71	Neurocan core protein
−5114.18	GTPase IMAP family member 4	−5076.28	Cytochrome c oxidase subunit 3	−712.63	Putative L-cysteine desulfhydrase 1
−3240.46	Hepatic lectin	−4899.42	Phosphoenolpyruvate carboxykinase	−693.30	Glyoxylate reductase/hydroxypyruvate reductase
−3109.94	Nacre apextrin-like protein 1	−4849.28	Bacterial hemoglobin	−620.87	Complement C1q-like protein 3
−2965.72	Nephrin	−3940.14	Betaine–homocysteine S-methyltransferase 1	−607.11	Metalloproteinase inhibitor 3
−2778.00	Neurocan core protein	−3754.37	Lysosome membrane protein 2	−596.49	Phosphate carrier protein
−2611.24	Sacsin	−3724.18	Multi-CRP-I 3	−562.79	Glyoxylate reductase/hydroxypyruvate reductase
−2395.37	Hemicentin-1	−3590.02	40S ribosomal protein SA	−510.31	Neuronal acetylcholine receptor subunit alpha-2
2410.00	TLD domain-containing protein 1	−3381.63	Venom allergen 5.01	−407.37	Probable cysteine protease RD21B
2436.01	Polyubiquitin	−3007.79	Vitellogenin-2	−246.99	Multi-CRP-I 3
2489.15	Hemicentin-1	−2451.55	Metalloproteinase inhibitor 3	−203.99	Hepatic lectin
2502.58	MAM and LDL-receptor class A domain-containing protein	−2405.10	Nacre apextrin-like protein 1	−198.48	Tubulin alpha chain
2725.96	D-arabinono-1,4-lactone oxidase	−2107.39	Myticin B	165.42	Sushi, nidogen and EGF-like domain-containing protein 1
3084.32	2′-5′-oligoadenylate synthase 1A	−1566.39	Papilin	197.54	C3a anaphylatoxin chemotactic receptor
3102.00	WAP four-disulfide core domain protein 2	−1482.80	Malate dehydrogenase, cytoplasmic	212.68	Mammalian ependymin-related protein 1
3139.69	Shell protein-5	−1466.81	Heme-binding protein 2	317.59	Proprotein convertase subtilisin/kexin type 5
3251.22	Galaxin	−1444.83	Stress-associated endoplasmic reticulum protein 2	336.24	Perlucin-like protein
3255.70	Netrin receptor DCC	−1435.92	Cytochrome c oxidase subunit 2	359.33	Peroxidasin homolog
3294.33	Nephrin	−1360.83	Defensin MGD-1	419.55	Spore cortex-lytic enzyme
4011.09	Spore cortex-lytic enzyme	−1283.57	Cytosolic 10-formyltetrahydrofolate dehydrogenase	427.42	Phenylalanine-4-hydroxylase
4027.72	Protein henna	−999.46	Glyceraldehyde-3-phosphate dehydrogenase	735.42	Protein henna
4927.82	Heat shock 70 kDa protein 12B	1357.30	Allene oxide synthase-lipoxygenase protein	915.43	Collagen alpha-2 (VIII) chain
5793.60	Low-density lipoprotein receptor-related protein 6	1451.53	Spore cortex-lytic enzyme	4103.11	Lysozyme

### Changes in the Response After the Second Encounter With the Same Pathogen

**Figure 4:**
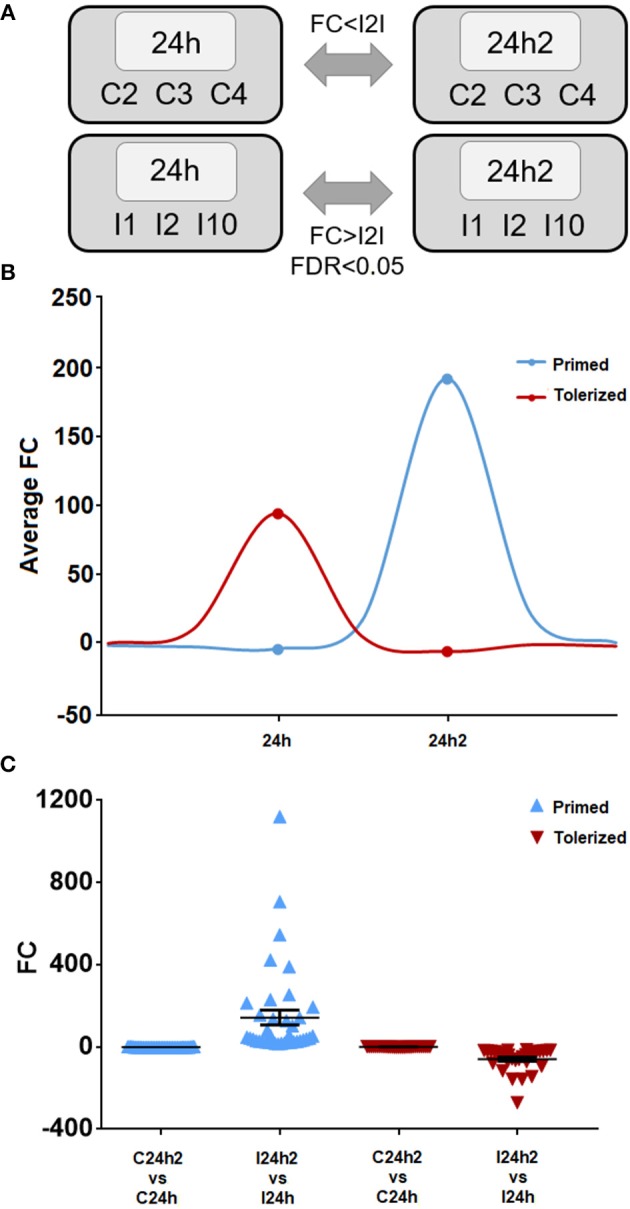
**(A)** Representative scheme of the comparisons made to select the primed and tolerized genes. The statistical parameters used as thresholds were an FDR < 0.05 and a fold change (FC) > |2| in the case of *Vibrio* injected individuals and a FC < |2| in control mussels. **(B)** Mean of the tendency of the primed and tolerized genes. **(C)** Fold change of each primed and tolerized gene and their respective controls. The mean and standard error of all represented genes is also shown.

Next, we looked for trainable genes with different expression values after reinjection compared to those after the first injection. We compared the transcriptomes of the challenged animals after the first and second exposures and selected primed genes, which were those with increased expression values after the second encounter with the same pathogen, or tolerized genes, which were those that showed decreased expression after reinjection (Figure 4A). A schematic representation of the expression behavior of these genes during the first and second injections with regard to naïve animals is shown in Figure 4B. Thirty-nine genes showed significantly increased expression after previous stimulation with *V. splendidus*, and 31 showed decreased expression. The expression levels of all these genes did not change in control animals after the first or second stimulation (Figure 4C).

The list of the annotated primed and tolerized DEGs is shown in Table 3. The regulation of these genes suggests an attenuation of inflammation, a decrease in radical oxygen species (ROS) production and the inhibition of apoptosis in the second contact with *V. splendidus*. Primed genes with increased expression in the second encounter, such as those encoding myomodulin neuropeptides, furin-like protease (KPC-1), and plasminogen/apolipoprotein(a), were directly or indirectly related to the control and inhibition of inflammatory processes. Moreover, there was high expression of genes involved in the inhibition of ROS, such as mitochondrial uncoupling protein 2-like (UCP2), oxidative stress-induced growth inhibitor 1 (OKL38), and NAD kinase. Finally, genes involved in the reduction of cell death (inhibitor of p53-induced apoptosis-beta) and DNA repair (DNA repair protein complementing XP-A cells) were also present in our set of primed genes. However, tolerized genes with a reduced expression level after the second encounter were associated with the activation of apoptosis and inflammatory response and included regulator of nonsense transcripts 1 (UPF1), nephrin, H/ACA ribonucleoprotein complex subunit 4 (DISKERIN), nuclear migration protein (nudC), and the dual serine/threonine and tyrosine protein kinase (RIP5).

**Table 3:** Identified primed and tolerized DEGs.

**Contig name**	**FC**	**Description**
**PRIMED DEGs 24h2 VS. 24h**		
Mg_contig_5596	423.00	Myomodulin neuropeptides
Mg_contig_14406	230.36	Mitochondrial uncoupling protein 2-like: UCP2
Mg_contig_1335	193.45	Tetraspanin-7
Mg_contig_9808	155.40	Mitochondrial uncoupling protein 2-like: UCP2
Mg_contig_4328	142.49	Furin-like protease: KPC-1
Mg_contig_30535	136.05	Tubulin beta chain
Mg_contig_14134	128.00	Plasminogen
Mg_contig_3665	57.18	Cytochrome P450 3A24
Mg_contig_50120	34.93	DNA repair protein complementing XP-A cells: XPA
Mg_contig_24689	30.88	Solute carrier family 12 member 8
Mg_contig_16026	30.15	Ropporin-1-like protein
Mg_contig_21973	27.24	Inhibitor of p53-induced apoptosis-beta
Mg_contig_13532	26.93	Zinc finger protein Eos
Mg_contig_3349	25.19	Oxidative stress-induced growth inhibitor 1: OKL38
Mg_contig_13357	23.18	Apolipoprotein(a)
Mg_contig_41851	22.52	Tetratricopeptide repeat protein 38
Mg_contig_36957	22.50	Hydrocephalus-inducing protein
Mg_contig_32198	22.28	Solute carrier family 12 member 8
Mg_contig_45429	18.20	Alpha-L-fucosidase
Mg_contig_15455	17.40	Solute carrier family 46 member 3
Mg_contig_13725	14.90	NAD kinase
**TOLERIZED DEGs 24h2 VS. 24h**		
Mg_contig_49351	−157.33	Usherin
Mg_contig_53728	−69.50	NFX1-type zinc finger-containing protein 1
Mg_contig_88014	−40.66	Regulator of nonsense transcripts 1: UPF1
Mg_contig_50460	−22.38	NFX1-type zinc finger-containing protein 1
Mg_contig_38773	−21.16	Nephrin
Mg_contig_1952	−19.36	H/ACA ribonucleoprotein complex subunit 4: DISKERIN
Mg_contig_2166	−18.87	Phenylalanine-tRNA ligase beta subunit
Mg_contig_39783	−17.22	Nuclear migration protein: nudC
Mg_contig_7966	−16.66	Phenylalanine-tRNA ligase alpha subunit B
Mg_contig_13367	−16.20	Dual serine/threonine and tyrosine protein kinase: RIP5
Mg_contig_14736	−13.65	Phenylalanine-tRNA ligase beta subunit

### Priming Induces the Modification of Functional Hemocyte Responses

**Figure 5:**
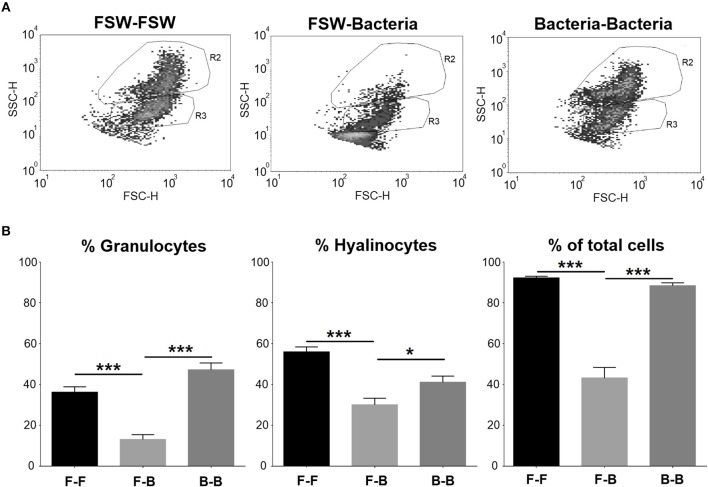
FACS analysis of the cell population distribution of mussel hemocytes after subsequent FSW/bacteria injection. **(A)** Dot plot of a total count of 100,000 events for hemolymph. R2, granulocytes; R3, hyalinocytes. **(B)** Statistical analysis of the hemocyte population in the different experimental conditions. Asterisks show the statistical significance: ^*^p < 0.05 and ^***^p < 0.0001. Subsequent stimulations are indicated as follows: F-F, FSW and FSW; F-B, FSW and bacteria; B-B, bacteria and bacteria.

Flow cytometry was used to better understand how two consecutive injections of *V. splendidus* affected mussel hemocytes. Two cell populations, granulocytes (R2) and hyalinocytes (R3), were well-defined in the control group (mussels injected with FSW at both sampling points) (Figure 5A). When mussels were injected with *V. splendidus*, the cell population structure was altered, and it was almost impossible to establish two separate populations of granulocytes and hyalinocytes. However, if mussels were stimulated with *V. splendidus* and received a second injection with the same pathogen, the hemocyte population structure was restored to that found in naïve mussels and showed a distribution similar to that in the controls. Quantitatively, the numbers of granulocytes and hyalinocytes were significantly reduced in mussels injected once with the bacteria compared to control animals. However, when mussels had been previously injected and received a second bacterial challenge, both cell types reached similar values to those found in the control (Figure 5B).

**Figure 6:**
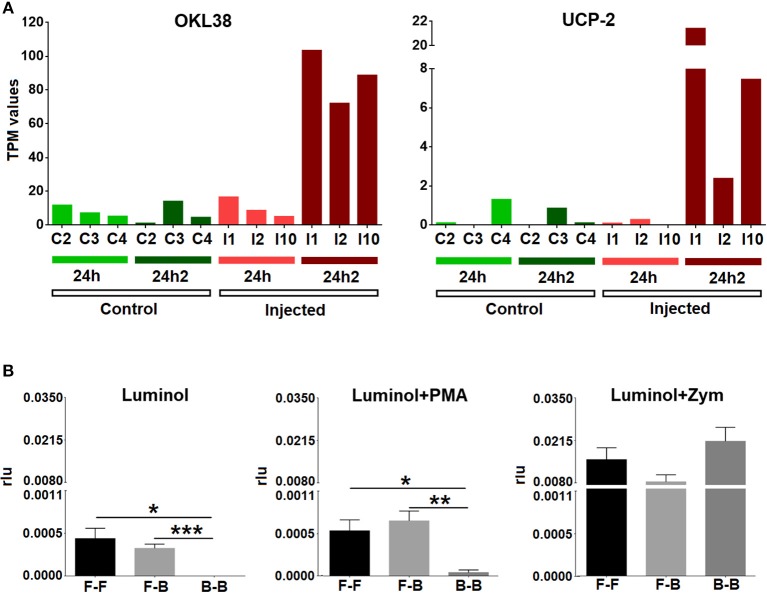
**(A)** Expression values (TPM) for two representative inhibitors of oxidative stress (OKL38 and UCP-2) in the six studied individuals (C2, C3, C4, I1, I2, and I10) at two experimental time points (24 hpi and 24 hpi2). **(B)** Respiratory burst assay. Bars represent the mean percentage and standard error of the relative luminescence units (rlu) for the total hemocytes in eight individual mussels. Asterisks show the statistical significance: ^*^p < 0.05; ^**^p < 0.001; and ^***^p < 0.0001. Subsequent stimulations are indicated as follows: F-F, FSW and FSW; F-B, FSW and bacteria; B-B, bacteria and bacteria.

To confirm the results of the transcriptomic analysis that suggested the inhibition of respiratory burst activity after the second injection, we looked closely at the expression values (TPM values) of some representative genes (OKL38 and UCP-2) in individual mussels. All challenged animals exhibited a significant increase in the expression of these two antioxidant genes after reinjection (24 hpi2) (Figure 6A). We also analyzed ROS production in hemocytes from treated mussels. Respiratory burst activity when there was no triggering molecule or that was triggered by PMA was notably decreased after the second injection (Figure 6B), supporting the results of the transcriptomic analysis. Respiratory burst activity mediated by zymosan A did not show significant differences among the three groups of mussels (FSW-FSW, FSW-bacteria, and bacteria-bacteria), which was probably due to the strong stimulating effect of zymosan A that masked the natural response (33).

**Figure 7:**
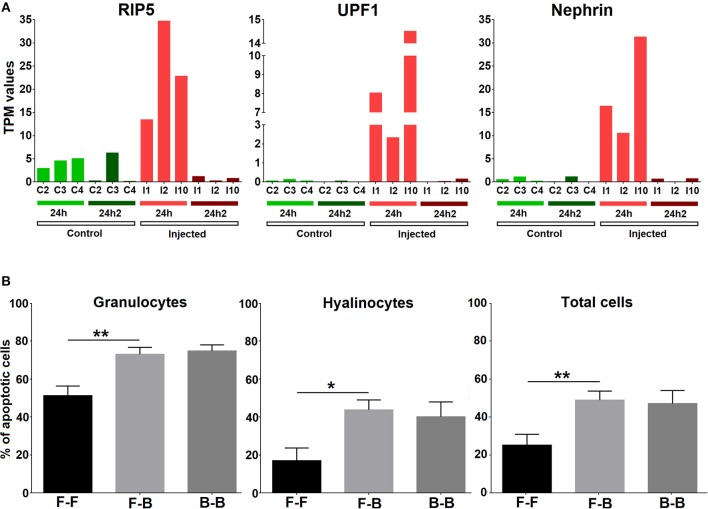
**(A)** Expression values (TPM) for three representative apoptosis inducers (RIP5, UPF1, and nephrin) in the 6 studied individuals (C2, C3, C4, I1, I2, and I10) at two experimental time points (24 hpi and 24 hpi2). **(B)** Apoptosis analysis. Bars represent the mean percentage and standard error of apoptotic cells in total hemocytes, hyalinocytes and granulocytes in eight individual mussels. Asterisks show the statistical significance: ^*^p < 0.05 and ^**^p < 0.001. Subsequent stimulations are indicated as follows: F-F, FSW and FSW; F-B, FSW and bacteria; B-B, bacteria and bacteria.

The transcriptomic analysis showed that apoptosis was another central process in the response that appeared to be strongly inhibited after the second *Vibrio* injection (24 hpi2); therefore, we analyzed the expression of cell death inducers such as UPF1, RIP5, and nephrin in individual mussels (Figure 7A) and confirmed the results observed in the global analysis. We next performed an experiment to analyze whether two subsequent challenges resulted in changes in cell death. The number of apoptotic granulocytes and hyalinocytes was significantly increased when mussels were injected with *V. splendidus*. However, if mussels were injected two consecutive times with a resting time in between, the number of apoptotic cells was similar to that detected after the first injection without any further increase in the apoptotic rate (Figure 7B).

**Figure 8:**
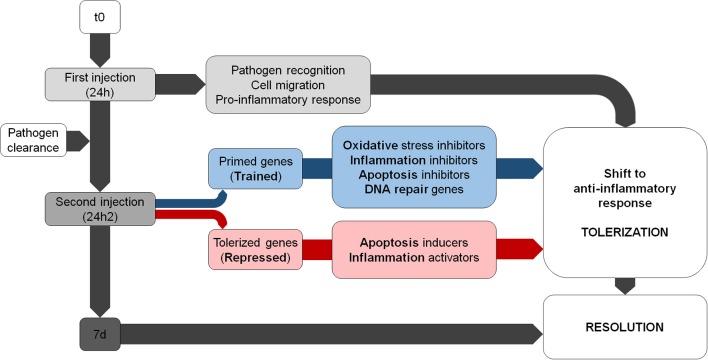
Summary of the main biological processes regulated during the subsequent *Vibrio* challenges. Note that the trained genes shift in terms of response from pro- to anti-inflammatory.

Our transcriptomic and functional results suggest that there is a modulation of the immune response after a second encounter with the same pathogen. Primed genes are involved in the resolution of the inflammatory process and the inhibition of ROS; however, repressed transcripts are related to inflammatory reactions and oxidative stress. There is a shift toward an anti-inflammatory response that attempts to minimize the damage caused by the second encounter with *V. splendidus* (Figure 8).

## Discussion

One of the characteristics of the innate immune system is its lack of immunological memory. However, in recent years, there is increasing evidence that innate immune cells can become reprogrammed to develop immunological memory after previous encounters with non-self-molecules (34–36). Bivalves, like all invertebrates, do not have an adaptive immune system and, due to their status as filter-feeding animals, are constantly in contact with microorganisms and environmental pollutants. Our results suggest that mussels may control the magnitude of their immune response, which allows them to deal with the continuous exposure to potential pathogens. A continuous reaction in these animals against all pathogenic and non-pathogenic microbes would potentially result in a constant state of inflammation that may be detrimental for the organism.

After the first *V. splendidus* exposure, there was an initial response represented by a high number of modulated genes, which decreased after the second exposure and almost returned to a basal level at the end of the experiment. The decrease in the number of differentially expressed genes after the second injection is in concordance with previous findings in *Crassostrea gigas* (37), in which specific protection against a viral infection was achieved after poly I:C priming and a later exposure to the pathogen did not trigger an antiviral response. This suggests that in our experimental design, the primary challenge with a pathogen triggered immune processes that could be reprogrammed afterwards.

When comparing the processes significantly enriched during the two subsequent encounters, a shift from an inflammatory to an anti-inflammatory state can be observed. After the first injection, the expected response would involve the positive regulation of the inflammatory response and the migration of hemocytes, as seen in other bivalves (10, 38). However, a strong decrease in the number of DEGs and the strict control of inflammation, which is a potentially harmful process, could be observed after the second challenge, which was possibly a consequence of the inhibition of the NF-kB signaling pathway (39). Taking into account that *Vibrio splendidus* was used at a sublethal dose and also that the infection was cleared before the second injection, it is unlikely that bacteria virulence factors have been responsible for the regulation of the inflammatory response. Also, we cannot know if the response after the second injection would be the same if a different pathogen was injected. These aspects should be further investigated.

Inflammation is critical in the response against infection; however, this process cannot last for a long time, and a return to a non-inflammatory state requires specific suppressor molecules (40). The expression of primed and tolerized genes in the mussel transcriptome suggests a combined response that attempts to control and limit three key immune processes: inflammation, ROS production, and apoptosis. The primed genes are involved in the attenuation of inflammation by regulating the transport of ions [myomodulin neuropeptides; (41, 42)], inhibiting NF-kB via ubiquitination [KPC-1; (43)], or inhibiting inflammatory pathways [plasminogen/apolipoprotein(a); (44–46)]. On the other hand, the tolerized genes (inhibited in the second challenge) are involved in the regulation of cell death [RIP5; (47)].

As is the case for the inflammatory process, an excess of reactive oxygen species can be harmful to the organism. At physiological levels, ROS are involved in intracellular signaling and defense, but uncontrolled production yields oxidative stress. Therefore, ROS are strictly controlled in all organisms to prevent self-inflicted damage (48, 49). The production of ROS is a well-characterized defense process in bivalves (50), but after the second exposure to the same pathogen, the mussels seemed to actively control oxidative stress by inhibiting respiratory burst activity with the expression of genes such as UCP2 (51), OKL38 (52), and NAD kinase (53). It seems that the control of oxidative stress is one of the central modulated processes after repeated encounters. Accordingly, at the functional level, we observed that the ROS levels were reduced after two subsequent *V. splendidus* challenges, suggesting that hemocytes could prevent an uncontrolled respiratory burst. Moreover, hemocytes seem to avoid cellular impairment caused by DNA damage resulting from previous oxidative stress (54), with the overexpression after the second injection of the DNA repair protein XPA (55).

A strong link between oxidative response and apoptosis, which is the other strongly modulated process in our results, has been reported (56, 57). The tolerized genes include several modulators of apoptosis. These genes, which have a predominantly pro-apoptotic function, are inhibited after the second exposure and include UPF1 (58), nephrin (59), DISKERIN (60), RIP5 (47), and nudC (61). At the functional level, the number of apoptotic cells was significantly increased after a single injection and was maintained, rather than increased, after the second injection.

In addition, the loss of the hemocyte population distribution after the first challenge and the restoration to normal conditions after the second challenge are in concordance with the presence of a priming process and reflect hemocyte recovery after the previous *V. splendidus* encounter. In our opinion, this evident change in hemocyte populations is very revealing and complements previous priming results in mollusks (62–65), and it should be further explored.

Eventually, 7 days after the second exposure, the resolution of the infection occurred. Processes related to tissue regeneration and learning are represented in our results, including the up-regulation of proliferation (C3a anaphylatoxin chemotactic receptor, skin secretory protein xP2, sushi, nidogen, and EGF-like domain-containing proteins), maintenance of the extracellular matrix (collagen, techylectin, perlucin, neurocan, and glycoproteins), and learning (phenylanaline-4-hydroxylase/henna protein). The presence of neural processes related to the generation of memory at all sampling points is remarkable. Although this might be due to the strong bias in the GO database in terms of model organisms, we cannot discard genuine evidence of a process that occurs during infection. The protein related to these GO terms, which is involved in the melanization cascade in invertebrates, could also be related to ancient cognition and behavior mechanisms, which are known to occur in invertebrates (66). In any case, it seems that certain processes, such as the metabolism of phenylalanine, could be involved in the generation of innate immune memory. Previous studies in mammals have shown the impairment of cognitive function due to phenylalanine hydroxylase deficiency (67–69), showing a possible link to learning processes and the evolutionary conservation of this mechanism.

In summary, the immune responses of *M. galloprovincialis* after the first and second encounter with *V. splendidus* were different. The analysis of the differentially expressed genes suggests that, after the second contact with the bacteria, the mussel hemocytes attempted to control and resolve the inflammatory response to avoid subsequent DNA damage and cell death. There appears a tightly regulated response shifting from a pro-inflammatory response to an anti-inflammatory and probably regenerative phenotype. In conclusion, these results indicate the existence of a secondary immune response in mussels oriented to tolerate infection by inducing anti-inflammatory processes to minimize tissue damage.

## Data Availability

The datasets generated for this study can be found in the NCBI Short Read Archive database under the accession ID SRP145077.

## Ethics Statement

The Mediterranean mussel, *M. galloprovincialis*, is not considered an endangered or protected species in any international species catalog, including the CITES list (www.cites.org), and it is not included in the list of species regulated by EC Directive 2010/63/EU. Therefore, no specific authorization is required to work on mussel samples.

## Author Contributions

BN and AF conceived and designed the project. RM and MR-C performed the mussel infections, sampling, and RNA extraction. AF, AP, MG, and RM performed the trimming, assembly, annotation and the gene expression, and statistical analyses. BN, MG, RM, and MR-C analyzed the generated data. MR-C performed the functional assays and wrote the manuscript. All listed authors revised, edited, read, and approved the manuscript.

### Conflict of Interest Statement

The authors declare that the research was conducted in the absence of any commercial or financial relationships that could be construed as a potential conflict of interest.

## Supplementary Material

Supplementary File 1 Complete list of contigs with BLAST description results, GO annotations, and KEGG enzymes.

Supplementary File 2 qPCR analysis to verify *V. splendidus* clearance 1 week after the challenge.

Supplementary File 3 DEGs after *V. splendidus* exposure at each sampling point, as determined by comparison with the controls.

## References

[1] Figueras A Biología y Cultivo de Mejillón (Mytilus galloprovincialis) en Galicia. Madrid: Consejo Superior de Investigaciones Científicas (2007). p. 284.

[2] Suttle CA. Marine viruses - major players in the global ecosystem. Nat Rev Microbiol. (2007) 5:801–12. 10.1038/nrmicro1750 17853907

[3] Farrington JW Tripp BW Tanabe S Subramanian A Sericano JL Wade TL Edward D. Goldberg's proposal of “the Mussel Watch”: reflections after 40years. Mar Pollut Bull. (2016) 110:501–10. 10.1016/j.marpolbul.2016.05.074 27339743

[4] Goldberg ED Bertine KK. Beyond the mussel watch-new directions for monitoring marine pollution. Sci Total Environ. (2000) 247:165–74. 10.1016/S0048-9697(99)00488-X 10803545

[5] Whitfield J. Vital signs. Nature. (2001) 411:989–90. 10.1038/35082694 11429567

[6] Garcia C Thébault A Dégremont L Arzul I Miossec L Robert M. Ostreid herpesvirus 1 detection and relationship with *Crassostrea gigas* spat mortality in France between 1998 and 2006. Vet Res. (2011) 42:73. 10.1186/1297-9716-42-73 21635731 PMC3129302

[7] Segarra A Pépin JF Arzul I Morga B Faury N Renault T. Detection and description of a particular Ostreid herpesvirus 1 genotype associated with massive mortality outbreaks of Pacific oysters, *Crassostrea gigas*, in France in 2008. Virus Res. (2010) 153:92–9. 10.1016/j.virusres.2010.07.011 20638433

[8] Domeneghetti S Varotto L Civettini M Rosani U Stauder M Pretto T. Mortality occurrence and pathogen detection in *Crassostrea gigas* and *Mytilus galloprovincialis* close-growing in shallow waters (Goro lagoon, Italy). Fish Shellfish Immunol. (2014) 41:37–44. 10.1016/j.fsi.2014.05.023 24909498

[9] Romero A Costa Md Forn-Cuni G Balseiro P Chamorro R Dios S. Occurrence, seasonality and infectivity of *Vibrio* strains in natural populations of mussels *Mytilus galloprovincialis*. Dis Aquat Organ. (2014) 108:149–63. 10.3354/dao02701 24553420

[10] Allam B Raftos D. Immune responses to infectious diseases in bivalves. J Invertebr Pathol. (2015) 131:121–36. 10.1016/j.jip.2015.05.005 26003824

[11] Costa MM Prado-Alvarez M Gestal C Li H Roch P Novoa B Functional and molecular immune response of Mediterranean mussel (*Mytilus galloprovincialis*) hemocytes against pathogen-associated molecular patterns and bacteria. Fish Shellfish Immunol. (2009) 26:515–23. 10.1016/j.fsi.2009.02.001 19340955

[12] Venier P Varotto L Rosani U Millino C Celegato B Bernante F. Insights into the innate immunity of the Mediterranean mussel *Mytilus galloprovincialis*. BMC Genomics. (2011) 12:69. 10.1186/1471-2164-12-69 21269501 PMC3039611

[13] Renault T Faury N Barbosa-Solomieu V Moreau K. Suppression substractive hybridisation (SSH) and real time PCR reveal differential gene expression in the Pacific cupped oyster, *Crassostrea gigas*, challenged with Ostreid herpesvirus 1. Dev Comp Immunol. (2011) 35:725–35. 10.1016/j.dci.2011.02.004 21371503

[14] Li J Zhang Y Zhang Y Liu Y Xiang Z Qu F. Cloning and characterization of three suppressors of cytokine signaling (SOCS) genes from the Pacific oyster, *Crassostrea gigas*. Fish Shellfish Immunol. (2015) 44:525–32. 10.1016/j.fsi.2015.03.022 25804492

[15] Wang Z Wang B Chen G Jian J Lu Y Xu Y. Transcriptome analysis of the pearl oyster (*Pinctada fucata*) hemocytes in response to *Vibrio alginolyticus* infection. Gene. (2016) 575:421–8. 10.1016/j.gene.2015.09.014 26363408

[16] Wei J Baosuo L Fan S Li H Chen M Zhang B. Differentially expressed immune-related genes in hemocytes of the pearl oyster *Pinctada fucata* against allograft identified by transcriptome analysis. Fish Shellfish Immunol. (2017) 62:247–56. 10.1016/j.fsi.2017.01.025 28126621

[17] Moreira R Balseiro P Forn-Cuní G Milan M Bargelloni L Novoa B Bivalve transcriptomics reveal pathogen sequences and a powerful immune response of the Mediterranean mussel (*Mytilus galloprovincialis*). Mar Biol. (2018) 165:61 10.1007/s00227-018-3308-0

[18] Jia Z Zhang H Jiang S Wang M Wang L Song L. Comparative study of two single CRD C-type lectins, CgCLec-4 and CgCLec-5, from pacific oyster *Crassostrea gigas*. Fish Shellfish Immunol. (2016) 59:220–32. 10.1016/j.fsi.2016.10.030 27765697

[19] Gerdol M Manfrin C De Moro G Figueras A Novoa B Venier P. The C1q domain containing proteins of the Mediterranean mussel *Mytilus galloprovincialis*: a widespread and diverse family of immune-related molecules. Dev Comp Immunol. (2011) 35:635–43. 10.1016/j.dci.2011.01.018 21295069

[20] Romero A Dios S Poisa-Beiro L Costa MM Posada D Figueras A. Individual sequence variability and functional activities of fibrinogen-related proteins (FREPs) in the Mediterranean mussel (*Mytilus galloprovincialis*) suggest ancient and complex immune recognition models in invertebrates. Dev Comp Immunol. (2011) 35:334–44. 10.1016/j.dci.2010.10.007 21034769

[21] Balseiro P Falcó A Romero A Dios S Martínez-López A Figueras A. *Mytilus galloprovincialis* myticin C: a chemotactic molecule with antiviral activity and immunoregulatory properties. PLoS ONE. (2011) 6:e23140. 10.1371/journal.pone.0023140 21858010 PMC3152575

[22] Novoa B Romero A Álvarez ÁL Moreira R Pereiro P Costa MM. Antiviral activity of myticin C peptide from mussel: an ancient defense against herpesviruses. J Virol. (2016) 90:7692–702. 10.1128/JVI.00591-16 27307570 PMC4988142

[23] Li H Parisi MG Toubiana M Cammarata M Roch P Lysozyme gene expression and hemocyte behaviour in the Mediterranean mussel, *Mytilus galloprovincialis*, after injection of various bacteria or temperature stresses. Fish Shellfish Immunol. (2008) 25:143–52. 10.1016/j.fsi.2008.04.001 18495491

[24] Gerdol M Venier P. An updated molecular basis for mussel immunity. Fish Shellfish Immunol. (2015) 46:17–38. 10.1016/j.fsi.2015.02.013 25700785

[25] Milutinovic B Kurtz J. Immune memory in invertebrates. Semin Immunol. (2016) 28:328–42. 10.1016/j.smim.2016.05.004 27402055

[26] Green TJ Montagnani C. Poly I:C induces a protective antiviral immune response in the Pacific oyster (*Crassostrea gigas*) against subsequent challenge with Ostreid herpesvirus (OsHV-1 μvar). Fish Shellfish Immunol. (2013) 35:382–8. 10.1016/j.fsi.2013.04.051 23685009

[27] Green TJ Helbig K Speck P Raftos DA. Primed for success: oyster parents treated with poly(I:C) produce offspring with enhanced protection against Ostreid herpesvirus type I infection. Mol Immunol. (2016) 78:113–20. 10.1016/j.molimm.2016.09.002 27616590

[28] Lafont M Petton B Vergnes A Pauletto M Segarra A Gourbal B. Long-lasting antiviral innate immune priming in the Lophotrochozoan Pacific oyster, *Crassostrea gigas*. Sci Rep. (2017) 7:13143. 10.1038/s41598-017-13564-0 29030632 PMC5640609

[29] Murgarella M Puiu D Novoa B Figueras A Posada D Canchaya C A First insight into the genome of the filter-feeder mussel *Mytilus galloprovincialis*. PLoS ONE. (2016) 11:e0151561 10.1371/journal.pone.0151561 26977809 PMC4792442

[30] Simão FA Waterhouse RM Ioannidis P Kriventseva EV Zdobnov EM. BUSCO: assessing genome assembly and annotation completeness with single-copy orthologs. Bioinformatics. (2015) 31:3210–2. 10.1093/bioinformatics/btv351 26059717

[31] McCarthy DJ Chen Y Smyth GK. Differential expression analysis of multifactor RNA-Seq experiments with respect to biological variation. Nucleic Acids Res. (2012) 40:4288–97. 10.1093/nar/gks042 22287627 PMC3378882

[32] Conesa A Götz S García-Gómez JM Terol J Talón M Robles M. Blast2GO: a universal tool for annotation, visualization and analysis in functional genomics research. Bioinformatics. (2005) 21:3674–6. 10.1093/bioinformatics/bti610 16081474

[33] Costa MM Novoa B Figueras A Influence of β-glucans on the immune responses of carpet shell clam (*Ruditapes decussatus*) and Mediterranean mussel (*Mytilus galloprovincialis*). Fish Shellfish Immunol. (2008) 24:498–505. 10.1016/j.fsi.2007.10.003 18329901

[34] Netea MG Quintin J van der Meer JWM. Trained immunity: a memory for innate host defense. Cell Host Microbe. (2011) 9:355–61. 10.1016/j.chom.2011.04.006 21575907

[35] Quintin J Cheng S-C van der Meer JWM Netea MG. Innate immune memory: towards a better understanding of host defense mechanisms. Curr Opin Immunol. (2014) 29:1–7. 10.1016/j.coi.2014.02.006 24637148

[36] Gourbal B Pinaud S Beckers GJM Van Der Meer JWM Conrath U Netea MG. Innate immune memory: an evolutionary perspective. Immunol Rev. (2018) 283:21–40. 10.1111/imr.12647 29664574

[37] Green TJ Benkendorff K Robinson N Raftos D Speck P. Anti-viral gene induction is absent upon secondary challenge with double-stranded RNA in the Pacific oyster, *Crassostrea gigas*. Fish Shellfish Immunol. (2014) 39:492–7. 10.1016/j.fsi.2014.06.010 24945571

[38] Rey-Campos M Moreira R Valenzuela-Muñoz V Gallardo-Escárate C Novoa B Figueras A. High individual variability in the transcriptomic response of Mediterranean mussels to Vibrio reveals the involvement of myticins in tissue injury. Sci Rep. (2019) 9:3569. 10.1038/s41598-019-39870-3 30837561 PMC6401078

[39] Ivanenkov YA Balakin KV Lavrovsky Y. Small molecule inhibitors of NF-kB and JAK/STAT signal transduction pathways as promising anti-inflammatory therapeutics. Mini Rev Med Chem. (2011) 11:55–78. 10.2174/138955711793564079 21034406

[40] Nathan C. Points of control in inflammation. Nature. (2002) 420:846–52. 10.1038/nature01320 12490957

[41] Tobin AE Calabrese RL. Myomodulin increases Ih and inhibits the NA/K pump to modulate bursting in leech heart interneurons. J Neurophysiol. (2005) 94: 3938–50. 10.1152/jn.00340.2005 16093342 PMC1560091

[42] Eisenhut M. Changes in ion transport in inflammatory disease. J Inflamm. (2006) 3:5. 10.1186/1476-9255-3-5 16571116 PMC1562419

[43] Kravtsova-Ivantsiv Y Kwon YT Ciechanover A Role of the ubiquitin ligase KPC1 in NF-κB activation and tumor suppression. J Anal Sci Technol. (2016) 7:8 10.1186/s40543-016-0087-4

[44] McLean JW Tomlinson JE Kuang WJ Eaton DL Chen EY Fless GM. cDNA sequence of human apolipoprotein(a) is homologous to plasminogen. Nature. (1987) 330:132–7. 10.1038/330132a0 3670400

[45] Ichinose A. Characterization of the apolipoprotein(a) gene. Biochem Biophys Res Commun. (1995) 209:365–71. 10.1006/bbrc.1995.1512 7726858

[46] Sulniute R Shen Y Guo YZ Fallah M Ahlskog N Ny L. Plasminogen is a critical regulator of cutaneous wound healing. Thromb Haemost. (2016) 115:1001–9. 10.1160/TH15-08-0653 26791370

[47] Zha J Zhou Q Xu LG Chen D Li L Zhai Z. RIP5 is a RIP-homologous inducer of cell death. Biochem Biophys Res Commun. (2004) 319:298–303. 10.1016/j.bbrc.2004.04.194 15178406

[48] Manduzio H Rocher B Durand F Galap C Leboulenger F The point about oxidative stress in molluscs. Invert Surviv J. (2005) 2:91–104.

[49] Bartosz G Reactive oxygen species: destroyers or messengers? Biochem Pharmacol. (2009) 77:1303–15. 10.1016/j.bcp.2008.11.009 19071092

[50] Donaghy L Lambert C Choi KS Soudant P Hemocytes of the carpet shell clam (*Ruditapes decussatus*) and the Manila clam (*Ruditapes philippinarum*): current knowledge and future prospects. Aquaculture. (2009) 297:10–24. 10.1016/j.aquaculture.2009.09.003

[51] Brand MD Esteves TC. Physiological functions of the mitochondrial uncoupling proteins UCP2 and UCP3. Cell Metab. (2005) 2:85–93. 10.1016/j.cmet.2005.06.002 16098826

[52] Li R Chen W Yanes R Lee S Berliner JA. OKL38 is an oxidative stress response gene stimulated by oxidized phospholipids. J Lipid Res. (2007) 48:709–15. 10.1194/jlr.M600501-JLR200 17192422

[53] Ying W. NAD+/NADH and NADP+/NADPH in cellular functions and cell death: regulation and biological consequences. Antioxid Redox Signal. (2008) 10:179–206. 10.1089/ars.2007.1672 18020963

[54] Bednarski JJ Sleckman BP. At the intersection of DNA damage and immune responses. Nat Rev Immunol. (2019) 19:231–42. 10.1038/s41577-019-0135-6 30778174 PMC6438741

[55] Sugasawa K. Xeroderma pigmentosum genes: functions inside and outside DNA repair. Carcinogenesis. (2008) 29:455–65. 10.1093/carcin/bgm282 18174245

[56] Simon HU Haj-Yehia A Levi-Schaffer F. Role of reactive oxygen species (ROS) in apoptosis induction. Apoptosis. (2000) 5:415–8. 10.1023/A:1009616228304 11256882

[57] Fleury C Mignotte B Vayssière JL. Mitochondrial reactive oxygen species in cell death signaling. Biochimie. (2002) 84:131–41. 10.1016/S0300-9084(02)01369-X 12022944

[58] Jia J Furlan A1 Gonzalez-Hilarion S Leroy C Gruenert DC Tulasne D. Caspases shutdown nonsense-mediated mRNA decay during apoptosis. Cell Death Differ. (2015) 22:1754–63. 10.1038/cdd.2015.18 25744026 PMC4648321

[59] Kapodistria K Tsilibary EP Politis P Moustardas P Charonis A Kitsiou P. Nephrin, a transmembrane protein, is involved in pancreatic beta-cell survival signaling. Mol Cell Endocrinol. (2015) 400:112–28. 10.1016/j.mce.2014.11.003 25448064

[60] Lin P Mobasher ME Alawi F. Acute dyskerin depletion triggers cellular senescence and renders osteosarcoma cells resistant to genotoxic stress-induced apoptosis. Biochem Biophys Res Commun. (2014) 446:1268–75. 10.1016/j.bbrc.2014.03.114 24690175 PMC4096983

[61] Zhou T Zimmerman W Liu X Erikson RL. A mammalian NudC-like protein essential for dynein stability and cell viability. Proc Natl Acad Sci USA. (2006) 103:9039–44. 10.1073/pnas.0602916103 16754861 PMC1482562

[62] Cong M Song L Wang L Zhao J Qiu L Li L. The enhanced immune protection of Zhikong scallop *Chlamys farreri* on the secondary encounter with *Listonella anguillarum*. Comp Biochem Physiol B Biochem Mol Biol. (2008) 151:191–6. 10.1016/j.cbpb.2008.06.014 18652907

[63] Zhang T Qiu L Sun Z Wang L Zhou Z Liu R. The specifically enhanced cellular immune responses in Pacific oyster (*Crassostrea gigas*) against secondary challenge with *Vibrio splendidus*. Dev Comp Immunol. (2014) 45:141–50. 10.1016/j.dci.2014.02.015 24607288

[64] Pinaud S Portela J Duval D Nowacki FC Olive MA Allienne JF. A shift from cellular to humoral responses contributes to innate immune memory in the vector snail *Biomphalaria glabrata*. PLoS Pathog. (2016) 12:e1005361. 10.1371/journal.ppat.1005361 26735307 PMC4703209

[65] Dubief B Nunes FLD Basuyaux O Paillard C. Immune priming and portal of entry effectors improve response to vibrio infection in a resistant population of the European abalone. Fish Shellfish Immunol. (2017) 60:255–64. 10.1016/j.fsi.2016.11.017 27836724

[66] Beets I Janssen T Meelkop E Temmerman L Suetens N Rademakers S. Vasopressin/oxytocin-related signaling regulates gustatory associative learning in C. elegans. Science. (2012) 338:543–5. 10.1126/science.1226860 23112336

[67] Hommes FA. Loss of neurotransmitter receptors by hyperphenylalaninemia in the HPH-5 mouse brain. Acta Paediatr Suppl. (1994) 407:120–1. 7766946 10.1111/j.1651-2227.1994.tb13469.x

[68] Gassió R Vilaseca MA Lambruschini N Boix C Fusté ME Campistol J. Cognitive functions in patients with phenylketonuria in long-term treatment with tetrahydrobiopterin. Mol Genet Metab. (2010) 99(Suppl. 1):S75–78. 10.1016/j.ymgme.2009.10.187 20123475

[69] Mitchell JJ Trakadis YJ Scriver CR. Phenylalanine hydroxylase deficiency. Genet Med. (2011) 13:697–707. 10.1097/GIM.0b013e3182141b48 21555948

